# Serum inorganic phosphorus levels predict 30-day mortality in patients with community acquired pneumonia

**DOI:** 10.1186/s12879-015-1094-6

**Published:** 2015-08-13

**Authors:** Mohammad E. Naffaa, Mona Mustafa, Mohje Azzam, Roni Nasser, Nizar Andria, Zaher S. Azzam, Eyal Braun

**Affiliations:** Department of Internal Medicine H, Rambam Health Care Campus, Haifa, Israel; B. Shine Rheumatology Unit, Rambam Health Care Campus, Haifa, Israel; Department of Internal Medicine E, Rambam Health Care Campus, Haifa, Israel; Department of Internal Medicine B, Rambam Health Care Campus, Haifa, Israel; The Rappoport’s Faculty of Medicine, Technion, Haifa, Israel; Department of Internal Medicine H and Infectious Diseases Unit, Rambam Health Care Campus, Haa’leya Hashneyya 8, Haifa, 31096 Israel

## Abstract

**Background:**

Community acquired pneumonia is a major cause of morbidity and mortality. The association between serum phosphorus levels on admission and the outcome of patients with community acquired pneumonia has not been widely examined. We aimed to investigate the prognostic value of serum phosphorus levels on admission on the 30- day mortality.

**Methods:**

The cohort included patients of 18 years old or older who were diagnosed with community acquired pneumonia between 2006 and 2012. Patients were retrospectively analyzed to identify risk factors for a primary endpoint of 30-day mortality. Binary logistic regression analysis was used for the calculation of the odds ratios (OR) and p values in bivariate and multivariate analysis to identify association between patients’ characteristic and 30-day mortality.

**Results:**

The cohort included 3894 patients. In multivariate regression analysis, variables associated with increased risk of 30-day mortality included: age >80 years, increased CURB-65 score, RDW >15, hypernatremia >150 mmol/l, hypoalbuminemia <2 gr/dl and abnormal levels of phosphorus. Levels of <1.5 mg/dl and >4.5 mg/dl were significantly associated with excess 30-day mortality, 38 % (OR 2.9, CI 1.8-4.9, P = 0.001) and 39 % (OR 3.4, CI 2.7-4.2, P = 0.001), respectively. Phosphorus levels within the upper normal limits (4-4.5 mg/dl) were associated with higher mortality rates compared to levels between 1.5-3.5 mg/dl, the reference group, 24 % (OR 1.9, CI 1.5-2.4, P = 0.001).

**Conclusions:**

Abnormal phosphorus levels on admission are associated with increased mortality rates among patients hospitalized with Community acquired pneumonia.

## Background

Community acquired pneumonia (CAP) is among the leading causes of mortality and severe morbidity especially among elderly population. Despite the efficacy of modern antibiotic treatment, it still ranks as the sixth most common cause of death [1–3]. Prognostic scores, like the CURB65 score and the Pneumonia Patient Outcomes Research Team score, were developed to estimate the risk of adverse outcome in patients treated in emergency rooms in an attempt to determine who is at risk for an adverse outcome, and therefore should be hospitalized [4, 5].

Phosphorus, as an essential component in the ATP molecule, plays a central role in the energy production . Serum phosphorus level disturbances in patients with pneumonia have been reported [6–9]. Hypophosphatemia is detected in 2-3 % of the patients hospitalized with medical illness [10–12]. Commonly reported etiologies for hypophosphatemia include alcohol abuse and withdrawal, diabetic ketoacidosis, nutritional recovery, alkalotic states, accelerated erythropoiesis and gram negative sepsis [13–19]. Many drugs have also been reported to cause hypophosphatemia, the most common being methylprednisolone, epinephrine, albumin, terbutaline, theophylline, and diethylsilbesterol [20].

Hypophosphatemia is known to play an essential role in impaired chemotaxis, phagocytosis, and bactericidal activity of macrophages [21]. Hypophosphtemia can lead to ATP depletion, a shift from oxidative phosphrylation toward glycolysis, and subsequently, organ dysfunction .and, especially, muscle weakness. Fisher et al found hypophosphatemia to be associated with longer hospital stay, but not with higher mortality in patients with respiratory illness [7]. Sankaran et al, on the other hand, reported that hypophosphatemic patients with pneumonia had longer hospital stay and higher mortality when compared with normophosphatemic patients [6].

In contrast to hypophosphatemia, the association between hyperphosphatemia and pneumonia has not been widely studied.Severe hyperphosphtemia may result in hypocalcemia which can cause tetany and pulmonary calcification. Saldias et al showed that hyperphosphatemia on admission represents a prognostic factor for in-hospital mortality in elderly patients with community acquired pneumonia [9].

In this cohort study, we aimed to examine the predictive prognostic value of serum phosphorus level on admission on the 30-day mortality in patients with community acquired pneumonia.

## Methods

Patients aged 18 years old or older who were diagnosed with CAP and admitted to Rambam Health Care Campus, a tertiary medical center, between 1 January, 2006 and 31 December, 2012 were retrospectively and consecutively analyzed to identify risk factors for 30-day mortality. CAP was defined as pneumonia identified within the first 48 hours of hospitalization. The diagnosis of pneumonia was confirmed when the patient fulfilled the criteria suggested by Fang [22]. These criteria are as follows: *a)* infiltrate in a chest x-ray taken on admission; *b)* the presence of one or more major findings (cough, mucopurulent or hemoptic expectoration, axillary temperature of over 37.8 °C); or *c)* at least two minor findings (pleuritic chest pain, dyspnea, decreased level of consciousness, lung tissue condensation observed in the physical lung examination, or a white blood count of over 12 000/mL). Protocol for treatment of CAP included either a combination of Ceftriaxone and Azithromycin or Levofloxacin as monotherapy. Data were collected from the Prometheus, an integrated computer system for handling patients’ medical records. The 30-day mortality data were retrieved from the database of our hospital and the ministry of health. Exclusion criteria included age under 18 years, transfer from another hospital, hospitalization during 30 days prior to admission, hospital-acquired pneumonia (defined as pneumonia which was diagnosed more than 48 hours after admission) or partial antibiotic treatment before hospitalization.

The following data were retrieved from the electronic medical records of the patients:

(1) Malignancies: solid tumors and hematologic malignancies. (2) Pulmonary diseases: bronchial asthma, chronic obstructive lung disease, interstitial lung disease, bronchiectasis, permanent tracheostomy, past history of thoracic radiotherapy, previous episode of pneumonia, and previous or current active smoker. (3) Immune suppression conditions: current chronic corticosteroid treatment, current or recent chemotherapy treatment, carrier of HIV, primary immune deficiency, history of bone marrow transplantation. (4) Cardiovascular diseases including patients with decompensated heart failure. (5) Chronic kidney disease including patients on dialysis. (6) Diabetes mellitus. (7) Liver cirrhosis. (8) Prior neurologic damage. (9) Chronic alcohol use. (10) Intravenous drug abuse. (11) Nursing house residents. The vital signs including heart rate, systolic blood pressure, respiratory rate, oxygen saturation and temperature were recorded on admission. The Charlson’s comorbidity index was calculated based on the data collected. The Charlson’s comorbidity index is a score that predicts the ten-year mortality for a patient who may have a range of comorbid conditions, (a total of 22 conditions), while each condition is assigned a score of 1, 2, 3, or 6, depending on the risk of dying associated with each condition. Scores are summed to provide a total score to predict mortality [23].

### Laboratory variables on admission

Serum glucose, serum creatinine, sodium, hemoglobin, white blood count, Red blood cell Distribution width (RDW), pH, calcium, phosphorus, bicarbonate, partial pressure of CO2, lactate, blood urea nitrogen (BUN), and serum albumin were measured on admission.

Hemoglobin levels, mean corpuscular volume and RDW were measured on admission, using the Advia 120 Hematology Analyzer (Siemens Healthcare Diagnostics Deerfield, Illinois, USA). Glucose, BUN and creatinine levels were measured using the “Dimension” (Siemens Healthcare Diagnostics Deerfield, Illinois, USA). The normal serum inorganic phosphorus range in the Rambam Health Care Campus laboratory is 2.5-4.5 mg/dl. Hypophosphatemia is defined as levels below 2.5 mg/d; whereas, levels above 4.5 mg/dl defines hyperphoshphatemia.

### Statistical analysis

Bivariate logistic regression analysis was used for the calculation of the odds ratios (OR) with 95 % Confidence Interval (CI) and *P* values in bivariate analysis to identify association between patient’s characteristic and 30-day mortality. Multivariate forward stepwise logistic regression was performed to assess the relation between patient’s characteristics: co-morbidities, laboratory results, and 30-day mortality.

Variables were selected as candidates for the multivariate analysis on the basis of the level of significance of the bivariate association with 30-day mortality (*P* < 0.1). Notably, there was no predilection in choosing serum phosphorus or any other variable in the statistical model.

The area under curve (AUC) was used as a measure of model of discrimination. The calibration of the prediction equation was assessed by comparing the observed and expected numbers of 30-day mortality. The Hosmer-Lemeshow goodness-of-fit statistic was calculated. We calculated the Spearman’s rank correlation coefficient to try to find out any correlation between variables that were found positive in the multivariate analysis. Two-tailed *P* values of 0.05 or less were considered as statistically significant. All statistical analyses were performed using SPSS (Statistics Products Solutions Services; Armonk, New York, USA) 21.0 software for Windows; Redmond, Washington, USA.

The Rambam Hospital Institutional Review Board approved the study. The approval number is 0515-12-RMB. The need for informed consent was waived.

## Results

Of the 5608 patients who were diagnosed with CAP in Rambam Health Care Campus between January 1, 2006 and December 31, 2012; 3876 patients had serum inorganic phosphorus levels were available within the first 24 hours of admission, and subsequently constituted our cohort. Of these 3876 patients, 57 % were males with median age of 69.6 years. The 30-day mortality was 17 % (*n* = 674). As shown in Table 1, the 30-day mortality was not significantly different between men and women. As well, the 30-day mortality each year was similar throughout the study period.

**Table 1:** Bivariate analysis of patients’ characteristics associated with 30-day mortality

Clinical RISK Factors		30 day-mortality				P-value	OR	95 % CI	
		Number	%	Number	%				
		3876	100 %	674	17 %				
Gender	Female	1664	43 %	282	17 %		Ref.		
	Male	2212	57 %	392	18 %	.529	1.056	.892	1.249
Age	<45	271	7 %	13	5 %	.000	Ref.		
	45-54	209	5 %	12	6 %	.252	1.490	.753	2.947
	55-64	356	9 %	30	8 %	.001	2.611	1.479	4.610
	65-74	579	15 %	74	13 %	.000	3.187	1.876	5.416
	75-84	1016	26 %	163	16 %	.000	5.387	3.247	8.936
	≥85	1445	37 %	382	26 %	.000	8.864	5.326	14.753
Year	2006-2008	1730	45 %	289	17 %	.230	Ref.		
	2009-2010	1058	27 %	202	19 %	.109	1.177	.965	1.435
	2011-2012	1088	28 %	183	17 %	.937	1.008	.823	1.235
Charson’s	No illnesses	599	15.4 %	57	9.5 %	.000	1		
Index	1	616	15.8 %	102	16.6 %	.000	2.511	1.625	3.882
	2	612	15.7 %	138	22.5 %	.000	3.361	2.203	5.129
	3-4	1105	28.4 %	326	29.5 %	.000	4.983	3.377	7.355
	5-7	723	18.6 %	245	33.9 %	.000	5.291	3.541	7.906
	8+	239	6.1 %	108	45.2 %	.000	8.678	5.522	13.637
CURB-65	0	589	15 %	11	2 %	.000	Ref.		
	1	881	23 %	76	9 %	.000	4.961	2.613	9.420
	2	1286	33 %	192	15 %	.000	9.222	4.981	17.073
	3	826	21 %	257	31 %	.000	23.733	12.838	43.875
	4	263	7 %	120	46 %	.000	44.094	23.157	83.959
	5	31	1 %	18	58 %	.000	72.755	27.708	184.387

Abbreviations: *OR* Odds Ratio, *CI* Confidence Interval, *Ref.* Reference

### Factors associated with 30-day mortality

As depicted in Table 1; 674 patients died within 30 days. Patients who died were older and had higher Charlson’s score reflecting more comorbid conditions. The year of diagnosis and inclusion did not influence the rate of 30-day mortality.

**Fig. 1:**
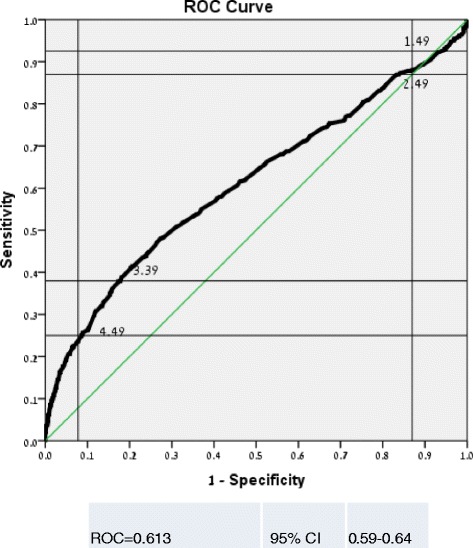
Serum Phosphrus ROC Curve

**Fig. 2:**
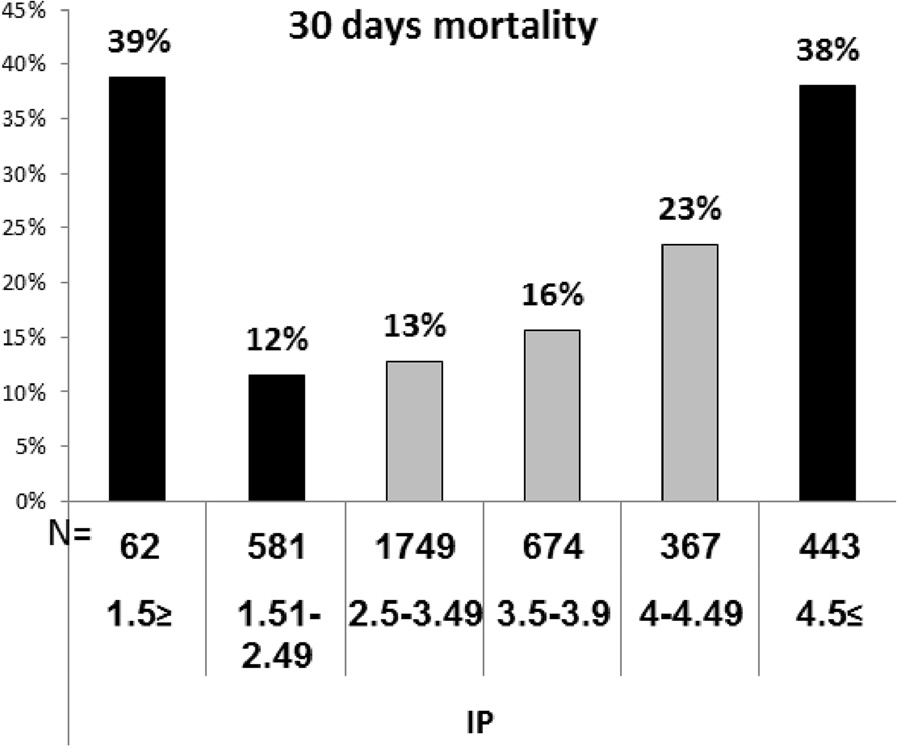
30-day mortality according to phosphorus levels

Table 2 shows the association between different laboratory parameters and 30-day mortality. When serum phosphorus levels were examined according to the normal laboratory range of our institution, that is, between 2.5 and 4.5 mg/dl and levels below 2.5 mg/dl representing hypophosphatemia and levels above 4.5 mg/dl representing hyperphosphatemia; only hyperphosphatemia, but not hypophosphatemia, was associated with increased mortality risk with odds ratio (OR), 95 % confidence interval (CI) and P value as follows: OR-3.5 (95 % CI 2.81-4.35, P < 0.0001). According to the ROC curve, cutoff levels of 1.51and 3.9 mg/dl were associated with significant change in specificity (Fig. 1). Therefore, we used levels between 1.51 and 3.9 as our new reference . Accordingly, the 30-day mortality rate was 13.2 % and increased to 38.7 % 30-day mortality rate with OR-4.16 (95 % CI 2.468-7.009, P <0.0001) in patient with levels ≤ 1.5 mg/dl. Notably, levels between 4-4.49 mg/dl and ≥ 4.5 mg/dl were associated with 23.4 % and 37.9 % 30-day mortality rate with OR-2.02 (95 % CI 1.548-2.6.24, P < 0.0001) and OR-4.02 (95 % CI 3.232-5.009, P < 0.0001), respectively. Figure 2 shows the correlation between different serum phosphorus levels and 30-day mortality.

**Table 2:** Bivariate analysis of laboratory parameters associated with 30-day mortality

		30 day- mortality				P-Value	OR	95 % CI	
Parameter		Number	%	N	%				
		3,876	100 %	674	17 %				
BUN (mg/dL)	<20	2754	71 %	298	11 %	.000	Ref.		
BUN (mg/dL)	20-39	485	13 %	134	28 %	.000	3.15	2.494	3.970
BUN (mg/dL)	40-59	382	10 %	117	31 %	.000	3.64	2.838	4.666
BUN (mg/dL)	≥60	255	7 %	125	49 %	.000	7.92	6.029	10.416
Creatinine (mg/dL)	0.9- 1.29	2258	58 %	288	13 %	.000	Ref.		
Creatinine (mg/dL)	0.1- 0.9	142	4 %	27	19 %	.034	1.606	1.037	2.486
Creatinine (mg/dL)	1.3-1.49	545	14 %	95	17 %	.004	1.444	1.121	1.861
Creatinine (mg/dL)	1.5-1.9	455	12 %	106	23 %	.000	2.078	1.618	2.668
Creatinine (mg/dL)	≥2	476	12 %	158	33 %	.000	3.399	2.708	4.266
Hemoglobin	≥12	1911	49 %	235	12 %	.000	Ref.		
Hemoglobin	10-11	1307	34 %	243	19 %	.000	1.664	1.404	1.972
Hemoglobin	9-10	380	10 %	99	26 %	.000	2.819	2.222	3.577
Hemoglobin	<9	269	7 %	95	35 %	.000	5.090	3.899	6.644
Hemoglobin	Missing	9		2	22 %				
Albumin (g/dL)	3.4-4	513	13 %	21	4 %	.000			
Albumin (g/dL)	<2	347	9 %	171	49 %	.000	22.76	14.018	36.963
Albumin (g/dL)	2-3	1668	43 %	313	19 %	.000	5.41	3.438	8.519
Albumin (g/dL)	3-3.4	721	19 %	55	8 %	.012	1.93	1.155	3.242
Albumin (g/dL)	Missing	627	16 %	114	18 %	.000	5.21	3.217	8.427
Sodium (mmol/L)	≤130	412	11 %	76	18 %	.000	1.00		
Sodium (mmol/L)	130-150	3366	87 %	543	16 %	.231	0.85	.652	1.109
Sodium (mmol/L)	≥150	97	3 %	55	57 %	.000	5.79	3.609	9.287
Sodium(mmol/L)	Missing	1		0	0 %				
WBC (10^3^/μL)	4 ≤ ≤12	1816	47 %	259	14 %	.000	Ref.		
WBC (10^3^/μL)	<4	144	4 %	27	19 %	.007	1.668	1.150	2.421
WBC (10^3^/μL)	>12	1907	49 %	386	20 %	.000	1.529	1.315	1.777
WBC (10^3^/μL)	Missing	9		2	22 %				
Hematocrit (%)	≥30	3268	85 %	496	15 %		Ref.		
Hematocrit (%)	<30	599	15 %	176	29 %	.000	2.735	2.280	3.280
Hematocrit (%)	Missing	9		2	22 %				
RDW (%)	≤15	1958	59 %	242	12 %		1.00		
RDW (%)	>15	1373	41 %	348	25 %	.000	2.41	2.008	2.886
RDW (%)	Missing	545		84	15 %				
GFR (ml/min)	≤90	852	22 %	106	12.4 %	.000	1.00		
GFR (ml/min)	60-90	1227	32 %	149	12.1 %	.839	0.97	.746	1.269
GFR (ml/min)	30-60	1332	34 %	260	19.5 %	.000	1.71	1.337	2.180
GFR (ml/min)	15-30	369	10 %	130	35.2 %	.000	3.83	2.850	5.141
GFR (ml/min)	<15	96	2 %	29	30.2 %	.000	3.05	1.883	4.927
Phosphorus (mg/dL)	2.5-4.49	2790	72 %	415	14.9 %	.000	1.00		
Phosphorus (mg/dL)	≤2.49	643	17 %	91	14.2 %	.641	0.94	.738	1.205
Phosphorus (mg/dL)	≥4.5	443	11 %	168	37.9 %	.000	3.50	2.810	4.350
Phosphorus (mg/dL)	1.51-3.9	3004	78 %	396	13.2 %	.000	1.00		
Phosphorus (mg/dL)	≤1.5	62	2 %	24	38.7 %	.000	4.16	2.468	7.009
Phosphorus (mg/dL)	4-4.49	367	9 %	86	23.4 %	.000	2.02	1.548	2.624
Phosphorus (mg/dL)	≥4.5	443	11 %	168	37.9 %	.000	4.02	3.232	5.009

Abbreviations: *OR* Odds Ratio, *CI* Confidence Interval, *Ref.* Reference, *BUN* Blood Urea Nitrogen, *WBC* White Blood Cells, *RDW* Red Blood Cell Distribution Width, *GFR* Glomerular Filtration Rate

### Relationship between Glomerular Filtration Rate (GFR), Phosphorus and Mortality

**Fig. 3:**
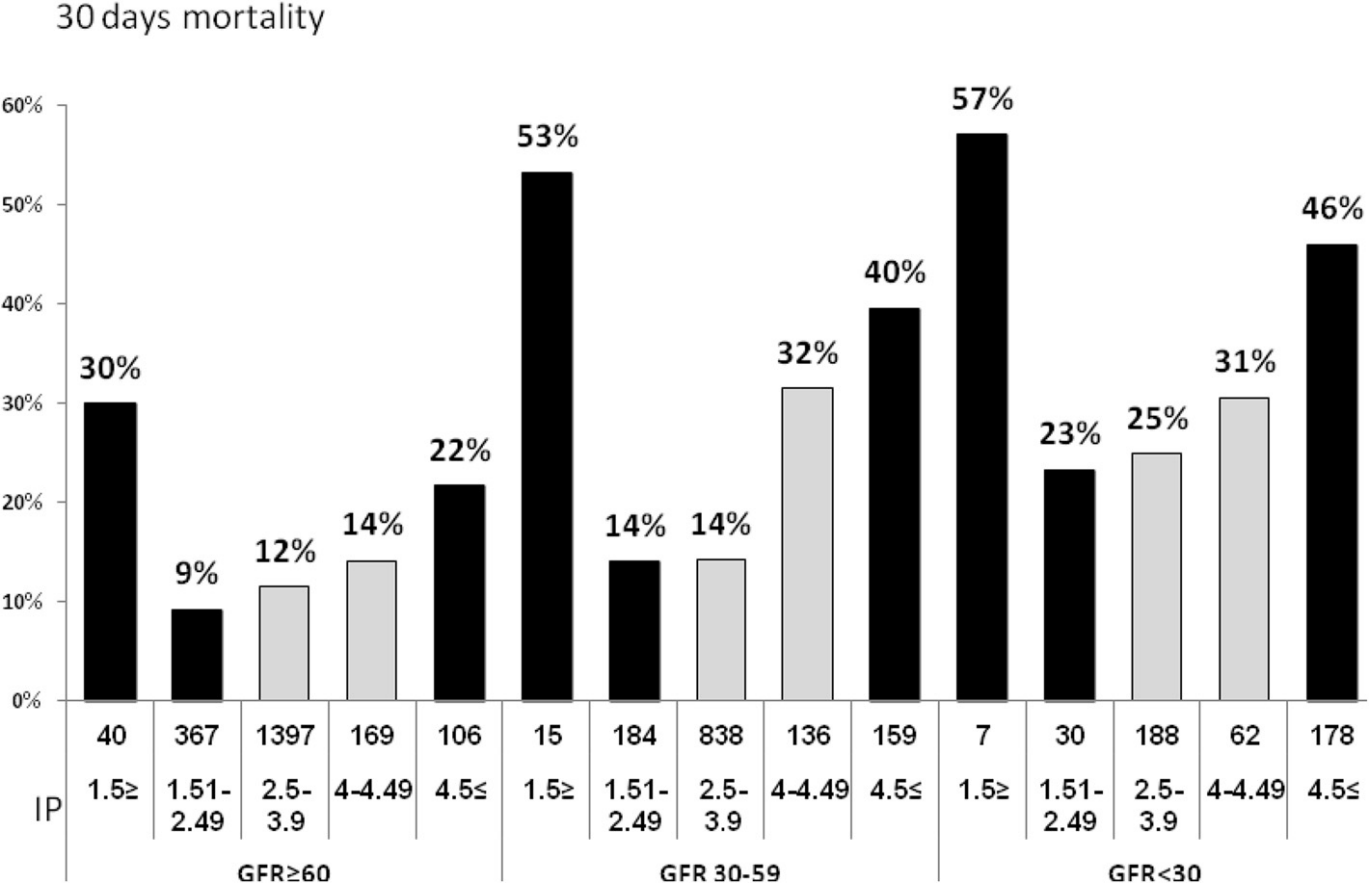
The relationship between GFR, Phosphorus and Mortality

As shown in Fig. 3, the predictive value of serum phosphorus levels on CAP outcome was maintained even after adjustment for GFR. Through all levels of GFR, hypophosphatemia and hyperphosphatemia were associated with increased mortality rates.

### Relationship between CURB-65, Phosphorus and Mortality

**Fig. 4:**
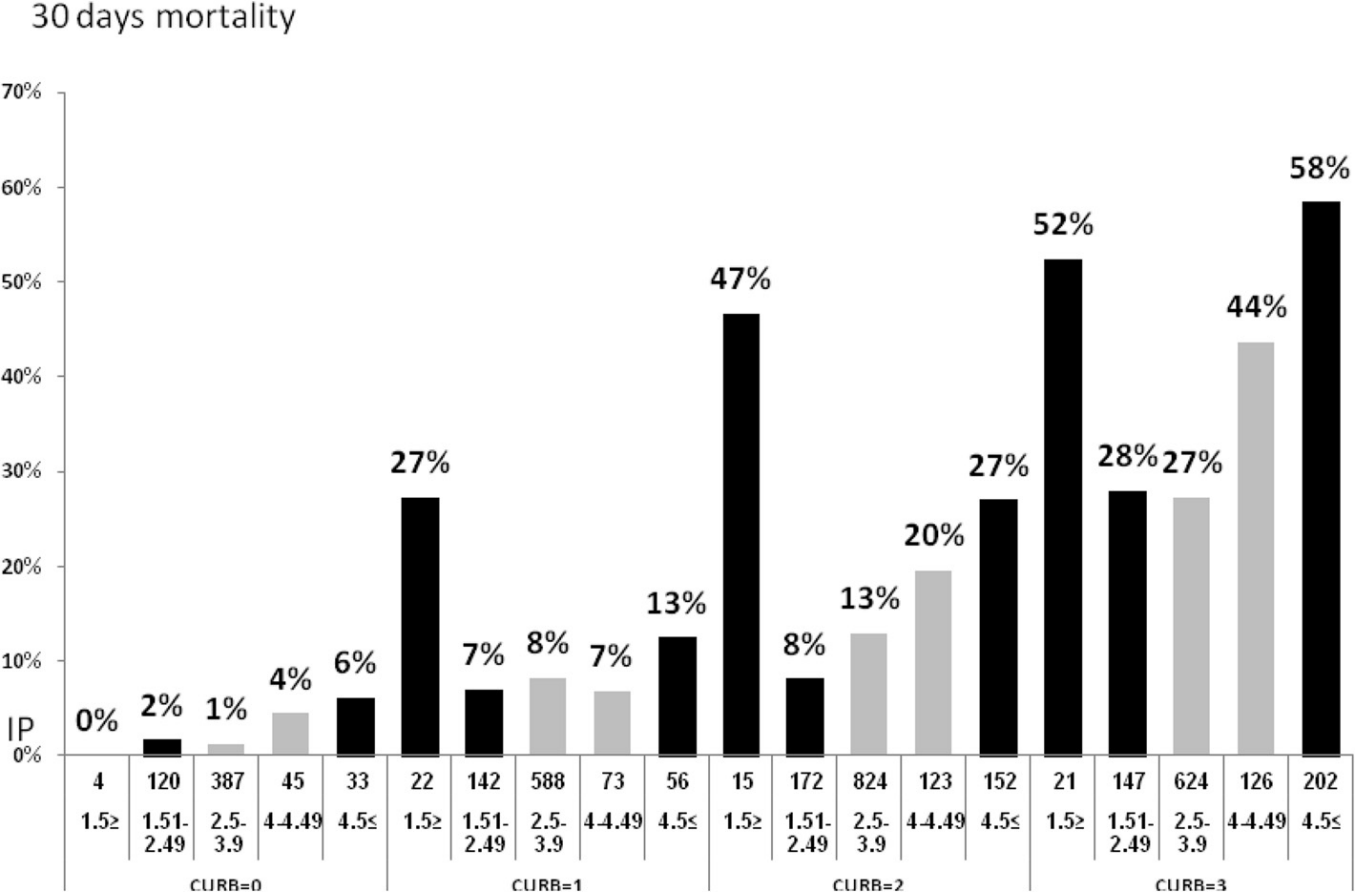
The relationship between CURB-65, Phosphorus and Mortality

The predictive value of serum phosphorus levels on CAP outcome was maintained after adjustment for CURB-65 score. While obviously higher CURB-65 score was associated with excess of mortality rates, severe hypophosphatemia and hyperphosphatemia were further associated with increased mortality rates for each CURB-65 score (Fig. 4).

### Relationship between Blood Urea Nitrogen (BUN), Phosphorus and Mortality

**Fig. 5:**
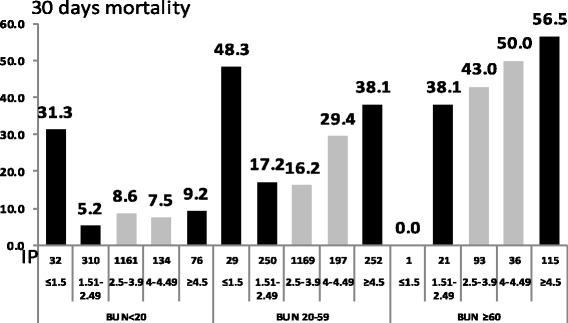
the relationship between BUN, Phosphorus and Mortality

As shown in Fig. 5, the predictive value of serum phosphorus levels on CAP outcome was maintained even after adjustment for BUN. Through all levels of BUN, hypophosphatemia and hyperphosphatemia were associated with increased mortality rates.

### Multivariate analysis of factors associated with 30-day mortality

As shown in Table 3, the following factors were associated with higher rates of 30-day mortality: sodium >150 meq/l, RDW > 15, low albumin levels (<2) and age >80 years. Increasing CURB-65 scores were associated with higher mortality. Whenever serum phosphorus levels were added to the model, severe hypophosphtemia (<1.5 mg/dl), levels between 4 and 4.5 mg/dl, and especially levels above 4.5 mg/dl were associated with significant mortality. The addition of serum phosphorus levels to the model improved AUC/ROC curve from 0.747 (95 % CI = 0.726-0.769) to 0.764 (95 % CI = 0.743-0.786). The Hosmer-Lemeshow goodness-of-fit statistic was not statistically significant (p = 0.77) indicating little departure and a perfect fit in both models.

**Table 3:** Multivariate analysis of factors associated with 30-day mortality

Parameter	Value	P-value	Adjusted OR	95 % CI	
				Lower	Upper
CURB-65	≤1	.000	Ref.		
	2	.017	1.4	1.1	1.9
	≥3	.000	3.5	2.6	4.7
Albumin (g/dL)	<2	.000	3.8	2.9	5.0
RDW(%)	>15	.000	1.6	1.3	1.9
Sodium (mmol/L)	≥150	.000	3.1	2.0	4.9
Age(years)	≥80	.000	1.6	1.3	2.0
Phosphorus (mg/dL)	1.51-3.9	.000	Ref.		
	≤1.5	.000	3.8	2.1	6.8
	4-4.49	.001	1.7	1.3	2.3
	≥4.5	.000	3.0	2.3	3.8

Abbreviations: *OR* Odds Ratio, *CI* Confidence Interval, *Ref.* Reference, *RDW* Red Blood Cell Distribution Width

In order to check for a possible correlation between serum phosphorus levels and other parameters, the sperman’s correlation coefficient was calculated; however no significant correlation was found (Table 4).

**Table 4:** Spearman’s Rank Correlation coefficient parameters

			% of Pts with P ≤ 1.5	% of Pts with P 1.51-2.49	% of Pts with P 2.5-3.99	% of Pts with P 4-4.49	% of Pts with P ≥4.5	SPEARMAN
Age (years)	<40	271	4	20	62	7	7	0.078
	40-49	209	2	15	65	11	7	
	50-59	356	1	21	58	8	11	
	60-69	579	1	16	62	9	11	
	70-79	1016	1	15	62	9	13	
	≥80	1445	2	12	64	10	12	
Albumin (g/dL)	3.4-4	513	0	12	65	11	12	0.040
	<2	347	4	17	50	10	18	
	2-3	1668	1	15	62	8	12	
	3-3.4	721	1	14	66	10	9	
	Missing	627	2	17	64	10	8	
Sodium (mmol/L)	≤130	412	2	19	61	7	12	0.049
	130-150	3366	2	15	63	10	11	
	≥150	97	1	12	55	13	19	
	Missing	1	0	100	0	0	0	
RDW (%)	≤15	1958	2	17	65	8	8	0.144
	>15	1373	1	11	59	11	17	
	Missing	545	2	17	64	10	7	
	60-90	1227	2	18	67	8	5	
	30-60	1332	1	14	63	10	12	
	15-30	369	2	7	44	14	33	
	<15	96	0	5	25	11	58	
CURB-65	0	589	1	20	66	8	6	
	1	881	2	16	67	8	6	
	2	1286	1	13	64	10	12	
	3	1120	2	13	56	11	18	

Abbreviations: *RDW* Red Blood Cell Distribution Width

## Discussion

In this study, we examined the role of serum phosphorus levels as a predictor of 30-day mortality in patients admitted to medical wards because of CAP. Our study demonstrated that serum phosphorus level obtained within 24 hours from admission can predict 30-day mortality, with levels below 1.5 mg/dl and levels above 4.5 mg/dl being associated with increased mortality levels. It is remarkable to note that when we followed the conventional international definitions for hyperphosphatemia (>4.5 mg/dl) and hypophosphatemia (<2.5 mg/dl), only hyperphosphatemia was associated with increased mortality. However, when levels between 1.5-3.9 mg/dl constituted our reference group with 30-day mortality of 13.2 %; levels below 1.5 mg/dl and above 4.5 mg/dl were both associated with increased 30-days mortality, 38.7 % and 37.9 %, respectively. Notably, even levels between 4-4.49 mg/dl, that are considered to be normal according to laboratory standards, were associated with 23.4 % 30-day mortality rates.

Phosphorus, as an essential component in the ATP molecule, plays a central role in the energy production. Therefore, depleted phosphorus stores, reflected by hypophosphatemia, might lead to insufficient and reduced ATP production which subsequently impairs several vital systemic functions including the immune system and the ability of the lungs to clear edema [24]. Craddock et al have shown that severe hypophosphatemia causes acquired phagocyte dysfunction reflected by defected chemotaxis, phagocytosis and bactericidal activity [21].

Hypophosphatemia in the setting of acute infectious illness such as CAP might have several etiologies, including refeeding, insulin therapy, acute respiratory alkalosis, inadequate intake, decreased phosphorus absorption (eg. anti-acids), and the use of medications (eg. methylprednisolone, epinephrine, terbutaline, and theophylline) [13–20]. On the contrary, the causes of hyperphosphatemia in the acute setting of CAP are very few and usually include acute renal failure, phosphorus-containing medications and lactic or ketoacidosis. Di Marco et al have reported that high phosphorus levels can impair endothelial cell function at several levels including induction of sustained stiffening, increased apoptosis, impaired angiogenesis, impaired cell migration, downregulation of extracellular annexin II expression and shedding of endothelial microparticles [25]. Altogether, this suggests that hyperphosphatemia can interfere with normal function of the immune system.

Our study demonstrated that increased serum creatinine and urea levels were also associated with increased 30-day mortality, therefore, we aimed to examine whether the association between serum phosphorus levels and 30-day mortality was related to renal failure. We reexamined the association between serum phosphorus levels and 30-day mortality after adjustment for GFR levels. As shown in Fig. 2, even after adjustment for GFR levels, serum phosphorus levels below 1.5 mg/dl and levels above 4 mg/dl were associated with increased 30-day mortality at each GFR subgroup. This indicates that serum phosphorus levels were associated with 30-day mortality regardless of GFR, creatinine or urea levels.

We also adjusted for CURB-65 score to evaluate whether serum phosphorus levels have an additional prognostic value. We showed that the prognostic value of serum phosphorus levels below 1.5 mg/dl and levels above 4 mg/dl was maintained at each CURB-65 score. Therefore, in levels below 1.5 mg/dl, intravenous treatment of elemental phosphuros should be strongly considered.

In this study we reproduced our previous findings showing the elevated RDW is associated with increased mortality in patients with Community acquired pneumonia [26].

Our study has several limitations. The first is the retrospective design of the study. Secondly, data regarding the exact cause of 30-day mortality was not available in all cases and unfortunately, chest radiography appearance on admission was not included among the parameters examined. The third limitation was that not all patients admitted with CAP had serum phosphorus levels within 24 hours from admission. This may, in fact, reflect the fact that serum phosphorus levels were available for the more severe patients. This fact is consistent with our finding that the predictive value of serum phosphorus levels was greater in higher CURB-65 scores. Because of the retrospective nature of the study, data regarding vitamin D levels, Parathyroid hormone, Fibroblast growth factor-23 (FGF-23) levels and the urinary phosphorus excretion were not available. These vitamins and hormones are known to play a central role in the hemostasis of serum phosphorus, and might subsequently affect 30-day mortality. A fourth limitation was the lack exact information about antibiotic treatment pre admission; therefore, unfortunately, these patients were excluded.

## Conclusions

Abnormal serum phosphorus levels on admission are associated with increased 30-mortality rates among adult patients hospitalized with CAP. The predictive value of phosphorus levels is maintained even after adjustment to GFR and CURB-65 levels.. We believe there is a real need to examine the prognostic predictive value of serum phosphorus levels on admission on 30-day mortality in patients with community acquired pneumonia prospectively, along with vitamin D levels, Parathyroid hormone, Fibroblast growth factor-23 (FGF-23) levels and the urinary phosphorus excretion.

## Abbreviations

CAP: Community acquired pneumonia
OR: Odds ratio
CI: Confidence interval
RDW: Red blood cell distribution width
GFR: Glomerular filtration rate
BUN: Blood urea nitrogen
